# Clinical Evaluation of Rega 8: An Updated Genotypic Interpretation System That Significantly Predicts HIV-Therapy Response

**DOI:** 10.1371/journal.pone.0061436

**Published:** 2013-04-17

**Authors:** Jurgen Vercauteren, Gertjan Beheydt, Mattia Prosperi, Pieter Libin, Stijn Imbrechts, Ricardo Camacho, Bonaventura Clotet, Andrea De Luca, Zehava Grossman, Rolf Kaiser, Anders Sönnerborg, Carlo Torti, Eric Van Wijngaerden, Jean-Claude Schmit, Maurizio Zazzi, Anna-Maria Geretti, Anne-Mieke Vandamme, Kristel Van Laethem

**Affiliations:** 1 Rega Institute for Medical Research, KU Leuven, Leuven, Belgium; 2 Northwest Institute of Bio-Health Informatics, University of Manchester, Manchester, United Kingdom; 3 Centro Hospitalar de Lisboa Ocidental, Lisbon, Portugal; 4 Retrovirology Laboratory, IRSICAIXA Foundation, Badalona, Spain; 5 Istituto di Clinica delle Malattie Infettive, Universita Cattolica del Sacro Cuore, Rome, Italy; 6 Sheba Medical Center, Tel-Hashomer, Israel; 7 Institute of Virology, University of Cologne, Cologne, Germany; 8 Division of Infectious Diseases, Department of Medicine, Karolinska Institute, Stockholm, Sweden; 9 Institute of Infectious and Tropical Diseases, University of Brescia, Brescia, Italy; 10 Department of Internal Medicine, University Hospitals Leuven, Leuven, Belgium; 11 Centre de Recherche Public de la Santé, Laboratory of Retrovirology, Luxembourg, Luxembourg; 12 Department of Molecular Biology, University of Siena, Siena, Italy; 13 Department of Virology, Royal Free Hampstead National Health Service Trust, London, United Kingdom; 14 Instituto de Higiene e Medicina Tropical, Universidade Nova de Lisboa, Lisbon, Portugal; 15 School of Public Health, Tel-Aviv University, Tel Aviv, Israel; University of Pittsburgh, United States of America

## Abstract

**Introduction:**

Clinically evaluating genotypic interpretation systems is essential to provide optimal guidance in designing potent individualized HIV-regimens. This study aimed at investigating the ability of the latest Rega algorithm to predict virological response on a short and longer period.

**Materials & Methods:**

9231 treatment changes episodes were extracted from an integrated patient database. The virological response after 8, 24 and 48 weeks was dichotomized to success and failure. Success was defined as a viral load below 50 copies/ml or alternatively, a 2 log decrease from the baseline viral load at 8 weeks. The predictive ability of Rega version 8 was analysed in comparison with that of previous evaluated version Rega 5 and two other algorithms (ANRS v2011.05 and Stanford HIVdb v6.0.11). A logistic model based on the genotypic susceptibility score was used to predict virological response, and additionally, confounding factors were added to the model. Performance of the models was compared using the area under the ROC curve (AUC) and a Wilcoxon signed-rank test.

**Results:**

Per unit increase of the GSS reported by Rega 8, the odds on having a successful therapy response on week 8 increased significantly by 81% (OR = 1.81, CI = [1.76–1.86]), on week 24 by 73% (OR = 1.73, CI = [1.69–1.78]) and on week 48 by 85% (OR = 1.85, CI = [1.80–1.91]). No significant differences in AUC were found between the performance of Rega 8 and Rega 5, ANRS v2011.05 and Stanford HIVdb v6.0.11, however Rega 8 had the highest sensitivity: 76.9%, 76.5% and 77.2% on 8, 24 and 48 weeks respectively. Inclusion of additional factors increased the performance significantly.

**Conclusion:**

Rega 8 is a significant predictor for virological response with a better sensitivity than previously, and with rules for recently approved drugs. Additional variables should be taken into account to ensure an effective regimen.

## Introduction

Since the advent of Highly Active Anti Retroviral Therapy (HAART), morbidity and mortality related with HIV/AIDS have considerably decreased in the Western world [1]. Clinicians can now compose several efficient combination regimens using 25 approved drugs [2]. Nevertheless, in many patients, not all options can be used, due to intolerance or side effects for certain drugs and because of the presence of antiviral drug (cross-)resistance [3]. Extended cross-resistance has been decreasing over calendar year and drug developers have been encouraged to focus their research on new potent drugs with a better tolerability, ease of use and less toxicity [4]. Nevertheless, resistance is and will continue to be an important issue in the management of HIV. Correct interpretation of the mutational patterns is however not straightforward, and unfortunately there is no consensus on this matter yet. Several genotypic interpretation systems have proven to significantly predict virologic response in retrospective analyses [5]–[7] and are therefore mentioned in treatment and resistance guidelines [8]. However, it remains a challenge to keep those interpretation systems up-to-date and improve their usefulness for clinicians treating HIV-infected patients [9]. New knowledge on resistance related mutations is accumulating and new drugs are still being implemented in clinical practice. Thus, guidelines stress the fact that regular updating and proper clinical evaluation of interpretation algorithms is needed [8].

The Rega algorithm was initiated in January 2000 and the current version Rega 8 dates from June 2009. Previous versions have been retrospectively evaluated with focus on short term viral response in HIV-1 patients (3 months) [6], [10]–[14]. Here the results of the clinical evaluation of Rega 8 for prediction of virologic response on short (8 weeks), mid-long (24 weeks) and long (48 weeks) term based on a large clinical database are presented, including a comparison with the HIVdb v6.0.11 and ANRS 2011.05 algorithms and a previous version of the Rega algorithm (Rega 5).

## Materials and Methods

An integrated database was set up using data originating from different countries: Belgium, Germany, Israel, Italy, Luxembourg, Portugal, Spain and Sweden, in collaboration with the EuResist consortium. This data didn’t serve as base for the construction of the rules-based interpretation systems discussed in this paper. RegaDB was used to manage and analyse the data in the integrated database [15], from which treatment change episodes (TCEs) were extracted. A TCE was defined as the start of a first line or follow-up therapy with the corresponding baseline variables and follow-up viral load measurements. A baseline PR-RT genotype and viral load measurement was required between 90 days before and one week after the start of the new therapy and at least one follow-up viral load measurement. Virological outcome of each TCE was assessed at 8, 24 and 48 weeks based on the latest viral load measurement. At 8 weeks, we defined virological success as the achievement of a viral load less than 50 copies/ml or a decrease from the baseline viral load by two or more Logs. At 24 and 48 weeks, the virological success was defined as the achievement of a viral load less than 50 copies/ml. No restrictions on therapies were contemplated, i.e. suboptimal treatment regimens made of less than three drugs were allowed. However TCEs containing inhibitors against other viral proteins than protease or reverse transcriptase were excluded because only PR-RT sequences were available. The following compounds, all currently approved by the Food and Drug Administration (FDA) (http://www.fda.gov/oashi/aids/virals.html) and European Medicines Agency (EMEA) (http://www.emea.europa.eu) were considered: nucleotide/side reverse transcriptase inhibitors (N(t)RTIs): zidovudine (AZT), stavudine (D4T), zalcitabine (DDC), abacavir (ABC), lamivudine (3TC), emtricitabine (FTC), didanosine (DDI), tenofovir (TDF); non-nucleoside reverse transcriptase inhibitors (NNRTIs): efavirenz (EFV), nevirapine (NVP), etravirine (ETR); protease inhibitors (PIs): amprenavir (APV), fosamprenavir (FPV), atazanavir (ATV), indinavir (IDV), lopinavir (LPV/r), nelfinavir (NFV), saquinavir (SQV), tipranavir (TPV), darunavir (DRV), along with boosting ritonavir (RTV). If multiple TCEs were available for a single patient, one was randomly selected to ensure a single TCE per patient. Based on previous reports [10], [13], potential confounding factors were included, if data on them was available: age, gender, risk group, baseline viral load and CD4 count, year of therapy start, introduction of a new drug class in the regimen, number of previous therapy switches and information on drug class experience (defined as more than 1 year on NRTIs, NNRTIs or PIs, respectively).

The viral genotypes were subtyped using version 2 of the Rega HIV-1 subtyping tool [16] and resistance interpretation was performed by Rega 5 (version 5.5; December 2001) and Rega 8 (version 8.0.2; June 2009) [14], version 6.0.11 (March 2011) of the Stanford HIVdb algorithm [17] and version 20 (May 2011) of the ANRS algorithm [18]. These algorithms (available as supplementary material) were used to assign a resistance score to each drug in the administered regimen. The Rega 5 algorithm and the ANRS algorithm consider 3 levels of resistance (corresponding with scores 0, 0.5, 1). The Stanford HIVdb algorithm considers 5 levels (0, 0.25, 0.5, 0.75 and 1). In the Rega 8 algorithm, the resistance scores are weighted based on the estimated potency and genetic barrier of the corresponding drug or drug class. The scores of boosted PIs are changed to 0, 0.75 and 1.5 and those for NNRTIs (except etravirine) become 0, 0.25 and 1 for resistant, intermediate resistant and susceptible respectively. Subsequently, the arithmetic sum of the scores for all drugs in the regimen was calculated to achieve the genotypic susceptibility score (GSS) of the regimen. Rules are removed from the algorithm when a drug is no longer used in clinical practice and new rules are implemented as soon as drug resistance mutations are known for a newly available drug. In order to compare Rega 5 and 8 on the same dataset, rules for an absent drug were copied from the other algorithm. Rules for boosted and un-boosted PIs were interchanged after correcting the weights. Rules for FTC and 3TC and for APV and its pro-drug FPV were interchanged. Full versions of the Rega algorithm can be found on the website of the KU Leuven http://regaweb.med.kuleuven.be/software/rega_algorithm/.

As the primary objective, the performance of Rega 8 was checked by univariate and multivariate logistic regression on the full dataset stratified for the three follow-up times and the odds ratio (OR), 95% confidence interval (CI) and p-values are reported. As a secondary objective, a comparison of Rega 8 with Rega 5, ANRS and Stanford HIVdb was performed. The individual performances were compared by means of a Receiver Operation Characteristic (ROC) analysis and Area Under the ROC Curve (AUC) was adopted by using 10-fold cross-validation. AUCs were then compared by using a Wilcoxon signed-rank test [19]. P-values were corrected for multiple testing using the Benjamini-Hochberg procedure. Additionally, the sensitivity was investigated for each individual algorithm using the GSS cut-off of 3 as suggested by recent guidelines [8]. Sensitivity (true positive rate) was defined as the ratio of the number of TCEs with a GSS of at least 3 and with virological success over the total number of virological successes. Specificity (true negative rate) was the ratio of the number of TCEs with a GSS less than the GSS cut-off and with virological failure over the total number of virological failures.

## Results

### Descriptive Statistics

**Figure 1:**
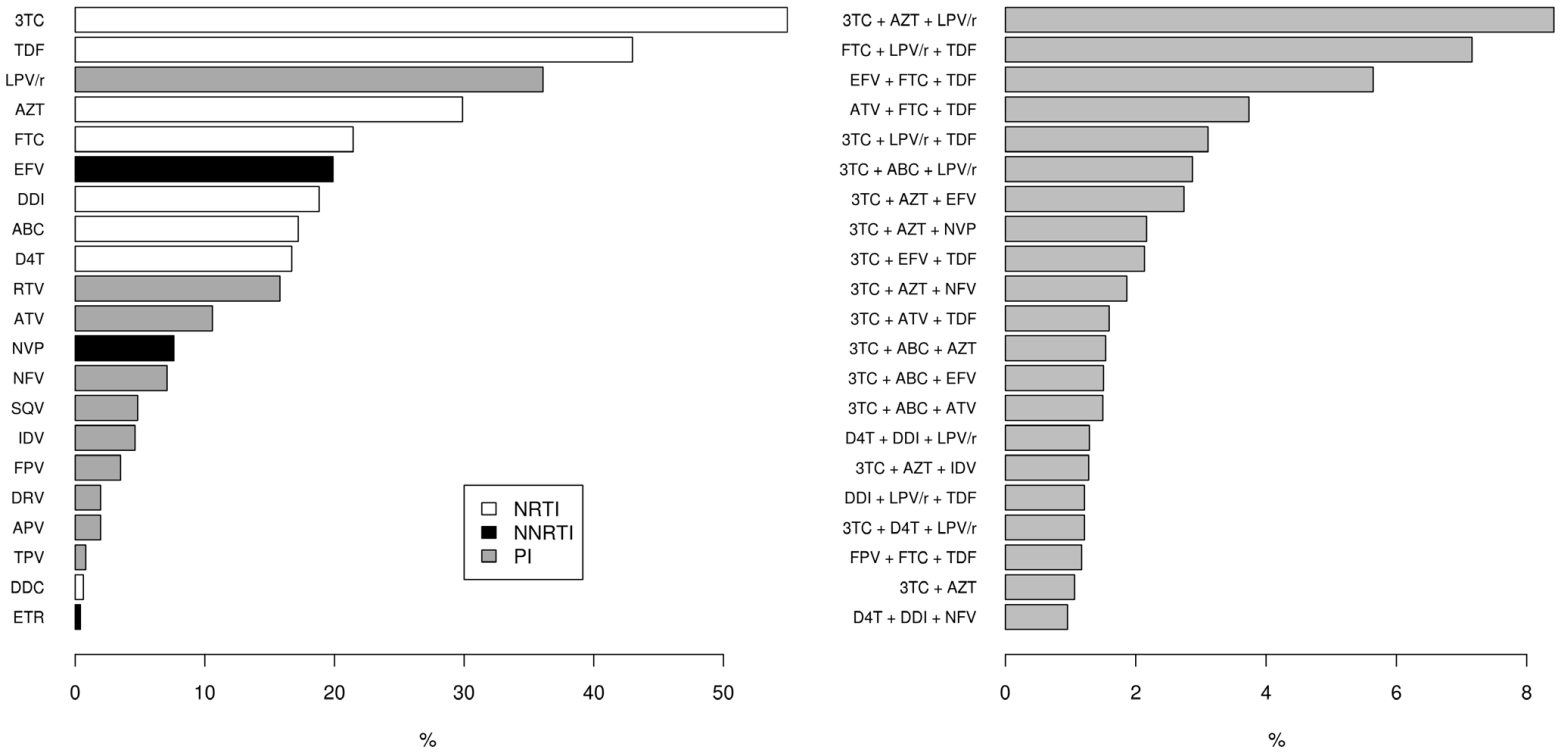
Prevalence rates (%) of antiretroviral drugs. Nucleotide/side reverse transcriptase inhibitors (N(t)RTIs): lamivudine (3TC), tenofovir (TDF), zidovudine (AZT), emtricitabine (FTC), didanosine (DDI), abacavir (ABC), stavudine (D4T), zalcitabine (DDC); non-nucleoside reverse transcriptase inhibitors (NNRTIs): efavirenz (EFV), nevirapine (NVP), etravirine (ETR); protease inhibitors (PIs): lopinavir (LPV/r), atazanavir (ATV), nelfinavir (NFV), saquinavir (SQV), indinavir (IDV), fosamprenavir (FPV), darunavir (DRV), amprenavir (APV), tipranavir (TPV), along with boosting ritonavir (RTV).

**Figure 2:**
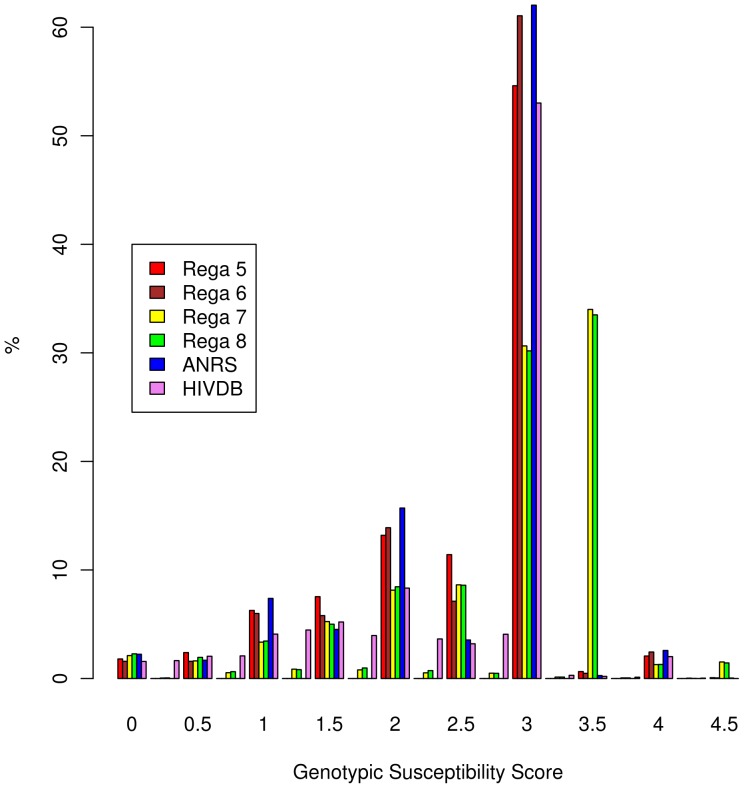
Distribution of the regimen-specific genotypic susceptibility scores reported by different versions of the Rega algorithm, ANRS v2011.05 and Stanford HIVdb v6.0.11.

9231 TCEs were extracted from the integrated database (Table 1). Median patient age was 40 years. 71% of patients were men, 35% were heterosexual, 26% were men who have sex with men and 20% were intravenous drug users. Most patients were infected with a subtype B virus (72%). Other prevalent 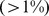 subtypes were subtype A (6%), subtype C (6%), CRF 02_AG (3%), subtype G (2%) and subtype F (2%). The ethnicity was unknown for most patients (69%). At baseline, the median CD4 cell count was 232 cells/mm^3^ and mean HIV-RNA load (viral load) 5.23 log_10_ copies/ml. 90% of the TCEs had a follow-up viral load measurement at 8 weeks, 93% at 24 and 82% at 48 weeks. The success rate dropped from 60% at 8 weeks to 50% at 24 weeks and to 47% at 48 weeks. The start date of the TCEs ranged from 1994 to 2010. 62% of the TCEs started for the first time with an NRTI, 8% with an NNRTI and 38% with a PI. The most prevalent drug administrated in the TCEs was 3TC, followed by TDF, LPV/r and AZT (Figure 1). At the start of the new regimen, experience for more than 1 year with an NRTI occurred in 53%, with an NNRTI in 23% and with a PI in 39% of the patients. The mean GSS reported by the algorithms ranged from 2.40 to 2.80 with standard deviations between 0.78 and 0.93 (Figure 2).

**Table 1:** Dataset characteristics.

Number of TCEs	9231	
Age, median (IQR), in years	40	11
Male, no. (%)	6378	71
Ethnicity, no. (%)		
Caucasian	1500	16
Asian	684	7
African	679	7
Unknown	6368	69
Risk group, no. (%)		
Heterosexual contact	3620	35
Men who have sex with men	2427	26
Injection drug use	1888	20
Transfusion	109	1
Vertical	124	1
Other	92	1
Unkown	1331	14
Subtype B, no. (%)	6649	72
Therapy start year, median (min-max)	2004	1994–2010
Follow-up time points, no. (%)		
At 8 weeks	8294	90
At 24 weeks	8566	93
At 48 weeks	7576	82
Success rate, no. (%)		
At 8 weeks	4937	60
At 24 weeks	4262	50
At 48 weeks	7576	47
New drug class in treatment, no. (%)		
NRTI	5769	62
NNRTI	721	8
PI	3465	38
Number of previous therapy switches, mean (SD)	4.15	4.02
Therapy experience^a^, no. (%)		
NRTI	4919	53
NNRTI	2139	23
PI	3590	39
Antiviral resistance^b^, no. (%)		
NRTI	4127	45
NNRTI	2300	25
PI	1905	21
Number of resistance mutations, mean (SD)		
NRTI	1.30	1.85
NNRTI	0.38	0.74
PI	0.42	0.97
Baseline CD4 cell count, cells/mm3, median (IQR)	232	260
Baseline HIV-RNA load, log_10_ copies/ml, mean(min-max)	5.23	0–7.54
Genotypic Susceptibility Score (GSS), median		
Rega 5	3	
Rega 8	3	
Stanford HIVdb v6.0.11	3	
ANRS v2011.05	3	

Characteristics of patients and treatment change episodes.

a At least 1 year of experience with a drug class.

b At least 1 resistance mutation from the IAS 2010 list.

### Performance of Rega 8

**Figure 3:**
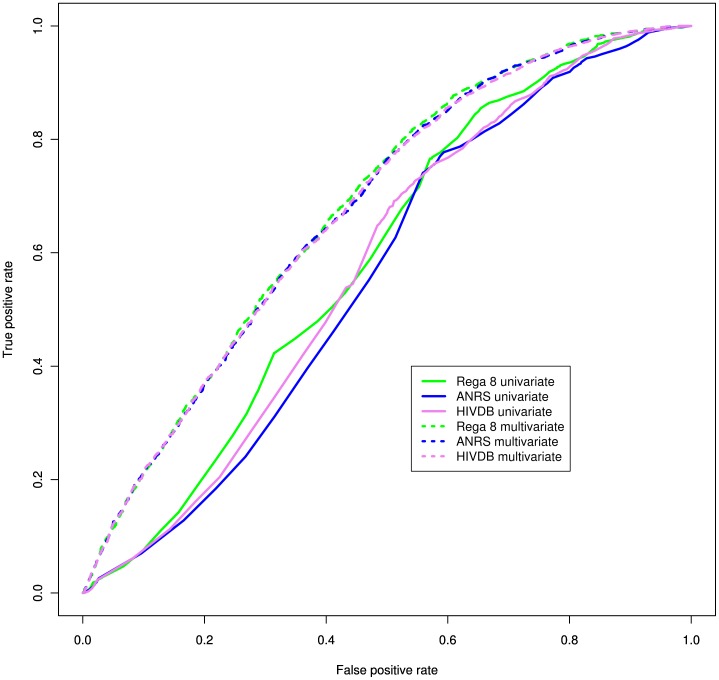
Receiver operating Characteristic (ROC) analysis of the 24-weeks-performance of the regimen-specific genotypic susceptibility score (GSS) according to Rega 8, ANRS v2011.05 and Stanford HIVdb v6.0.11 algorithms.

The odds ratios, 95% confidence intervals and p-values of the logistic regression models were determined for 8, 24 and 48 weeks response using the univariate approach (Table 2). The GSS as reported by Rega 8 was predictive for the virological success at all time-points. Per unit increase of the GSS, the odds on having a successful therapy response on week 8 increased by 81% (OR = 1.81, CI = [1.76–1.86], 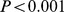), on week 24 by 73% (OR = 1.73, CI = [1.69–1.78], 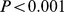) and on week 48 by 85% (OR = 1.85, CI = [1.80–1.91], 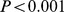) (Table 2). The ROC analysis of the 10-fold cross-validation showed that the inclusion of all available covariates significantly increased the performance of Rega 8 on all time points (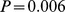, 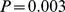 and 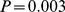 for week 8, 24 and 48 respectively). Also ANRS and HIVdb improved significantly when adding covariates to the model (Figure 3 for 24 weeks results).

**Table 2:** Performance on the complete dataset.

			logistic model			Area under the ROC curve		
approach	time	algorithm	OR	CI	P	Median	SD	Comparison*
univariate	8 weeks	Rega 5	1.95	1.89–2.01	<0.001	0.617	0.025	0.145
		Rega 8	1.81	1.76–1.86	<0.001	0.607	0.036	
		Rega 8 unweighted	1.90	1.85–1.96	<0.001	0.616	0.025	0.240
		ANRS	1.88	1.83–1.94	<0.001	0.608	0.021	1.000
		Stanford HIVdb	1.89	1.84–1.95	<0.001	0.626	0.030	0.079
	24 weeks	Rega 5	1.80	1.75–1.86	<0.001	0.598	0.037	0.782
		Rega 8	1.73	1.69–1.78	<0.001	0.601	0.045	
		Rega 8 unweighted	1.75	1.70–1.80	<0.001	0.596	0.038	0.372
		ANRS	1.74	1.69–1.79	<0.001	0.592	0.036	0.221
		Stanford HIVdb	1.69	1.64–1.73	<0.001	0.597	0.039	0.330
	48 weeks	Rega 5	1.95	1.89–2.02	<0.001	0.615	0.040	0.578
		Rega 8	1.85	1.80–1.91	<0.001	0.619	0.050	
		Rega 8 unweighted	1.86	1.80–1.91	<0.001	0.608	0.039	0.145
		ANRS	1.82	1.76–1.88	<0.001	0.603	0.038	0.088
		Stanford HIVdb	1.81	1.75–1.86	<0.001	0.612	0.042	0.240
multivariate	8 weeks	Rega 8	1.50	1.46–1.55	<0.001	0.653	0.036	
		ANRS	1.52	1.47–1.57	<0.001	0.652	0.037	0.913
		Stanford HIVdb	1.55	1.50–1.60	<0.001	0.655	0.038	0.555
	24 weeks	Rega 8	1.54	1.49–1.59	<0.001	0.667	0.050	
		ANRS	1.53	1.47–1.58	<0.001	0.663	0.050	0.162
		Stanford HIVdb	1.49	1.45–1.55	<0.001	0.663	0.049	0.145
	48 weeks	Rega 8	1.55	1.50–1.60	<0.001	0.680	0.054	
		ANRS	1.50	1.45–1.56	<0.001	0.675	0.056	0.145
		Stanford HIVdb	1.51	1.46–1.56	<0.001	0.676	0.054	0.145

Overview of the performance of the different algorithms using the univariate and multivariate approach and on 8, 24 and 48 weeks of therapy. The multivariate approach includes additional variables in the model: start year of therapy, information on start of a new drug class, number of previous therapy switches, previous drug class experience, baseline viral load, baseline CD4, gender, age, risk group. Reported are the odds ratio (OR), 95% confidence interval (CI) and P-value (P) of the logistic model and the median and standard deviation (SD) of the 10-fold cross-validation area under the ROC curve (AUC).

* The performance of the algorithms is compared with that of Rega 8 using a Wilcoxon signed-rank test and the P-value corrected for multiple testing is reported.

### Comparison with Rega 5, ANRS and Stanford HIVdb Algorithms

Rega 8 and Rega 5 performed equally well in predicting virological success on all time-points as the AUC was not statistically different: 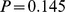, 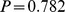 and 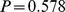 for week 8, 24 and 48 respectively (Table 2). However, Rega 8 had a higher sensitivity than Rega 5∶76.9% versus 67.4%, 76.5% versus 66.6% and 77.2% versus 67.4% on 8, 24 and 48 weeks respectively (Table 3). Without applying weights to the rules in Rega 8, the sensitivity decreased to 73.4%, 72.5% and 73.2% on 8, 24 and 48 weeks respectively.

**Table 3:** Sensitivity and Specificity.

	8 weeks		24 weeks		48 weeks	
	Sens	Spec	Sens	Spec	Sens	Spec
Rega 5	67.4	54.2	66.6	51.1	67.4	54.3
Rega 8	76.9	45.6	76.5	43.2	77.2	45.8
Rega 8 unweighted	73.4	49.1	72.5	46.1	73.2	48.6
ANRS	74.5	46.6	74.1	44.1	74.8	46.6
HIVDB	65.8	56.6	64.1	52.8	64.9	55.6

Sensitivity and specificity of the different algorithms after 8, 24 and 48 weeks of therapy using a cut-off GSS of 3. The sensitivity was defined as the proportion of TCEs with a GSS of 3 or more and a virological response on all those with a virological response whereas the specificity was seen as the proportion of TCEs with a GSS less than 3 and no virological response on all those with no virological response.

The performance of Rega 8 was further compared to those of the ANRS and Stanford HIVdb algorithms. No significant differences in AUC were found after correcting for multiple testing (Table 2). Both ANRS and Stanford HIVdb had a lower sensitivity than Rega 8: respectively 74.5% and 65.8% compared to 76.9% on 8 weeks, 74.1% and 64.1% compared to 76.5% on 24 weeks and 74.8% and 64.9% compared to 77.2% on 48 weeks.

## Discussion

In this study the Rega HIV drug resistance interpretation algorithm version 8 was retrospectively evaluated and compared with other systems using *in vivo* data from 9231 patients. We applied the algorithm to the baseline genotype and evaluated the association with the follow-up virologic response. Virological suppression means undetectable viral load but this threshold may differ according to the period in which measurements were done, with the newer viral load assays having a cut-off of 50 copies/ml. Excluding all measurements with a cut-off different from 50 copies/ml was difficult as we had a large dataset from various countries and laboratories that updated their assays at different times. However, we included the start date of therapy in the multivariate analysis to correct (partly) for those differences in assays.

The studied algorithms, Rega, ANRS and HIVdb, were able to significantly predict virological response of the TCEs. On average, per unit increase of the genotypic susceptibility score of the administrated regimen, the odds on having a successful therapy response almost doubled. Inclusion of treatment history and baseline patient- and viral derived characteristics enhanced the predictive value of the model. However these factors are not always available to the clinician nor are they automatically taken into account by the online available interpretation systems.

The predictive value reported here can only be a combined evaluation of the rules for those drugs that are present in all algorithms. Rega 5 contains rules for six NRTIs (AZT, DDC, DDI, 3TC, D4T, ABC), one NtRTI (TDF), three NNRTIs (NVP, DLV, EFV) and six PIs (RTV, IDV, SQV, NFV, APV, LPV/r). Rega 8 differs from Rega 5 in respect to drugs included: one NRTI (FTC), one NNRTI (ETV), four boosted PIs (FPV(r), ATV(r), TPVr and DRVr), two entry inhibitors (T20 and MVC) and one integrase inhibitor (RAL) are in Rega 8 and not in Rega 5. DDC is no longer in Rega 8 (discontinued since 2006), whereas the rules for APV are replaced by the ones for its prodrug FPV(r). Furthermore, existing rules are updated, a score-based system for PIs is implemented instead of logical rules and drug weighting factors are incorportated. Finally, Rega 8 has specific rules for the interpretation of HIV-2 drug resistance (which were not evaluated in this study). We saw that these differences between Rega 8 and 5 didn’t influence significantly the performance of the Rega algorithm in predicting therapy outcome. Moreover, Rega 8, ANRS and HIVdb reached the same performance on all time-points and using the univariate as well as the multivariate approach. In this discussion, we explore some of the possible reasons for this.

Our paper confirms the results of colleagues Frentz *et al.* [20] to that extent that the three commonly used HIV drug resistance interpretation systems - ANRS, Rega and HIVdb - predict virological response to the same extent. However, our paper evaluates more recent versions of the algorithms that are currently being used in clinical practice. Moreover it compares the latest Rega algorithm (version 8) with the previous evaluated version 5 to check consistency and improvement. Finally, it is for the first time that Rega, ANRS and Stanford HIVdb algorithms were evaluated on such an extensive dataset: 9231 TCEs from patients from several European countries. However, we have to acknowledge that the dataset still has its limitations. Despite the fact that the Rega algorithm is set up to be used for all HIV subtypes, our dataset contained 72% subtype B strains (Table 1), while the other subtypes were underrepresented [21]. Additionally, there is a bias present in the administered drugs: NRTIs that are commonly used to form the backbone regimen are highly prevalent in the dataset (e.g. 3TC was part of the regimen in almost half of the the cases), while more recently approved drugs (e.g. ETR) are underrepresented (Figure 1). Moreover, the prevalence of resistance mutations in the dataset follows a similar distribution with NRTI resistance being more prevalent than NNRTI or PI resistance (Table 1). This means that studies investigating the performance of interpretation systems specifically for recently approved drugs, less prevalent mutations and non-B subtypes are needed [9]. Moreover, only PR or RT genotypes were available so drugs targeting other regions had to be excluded from the dataset and the corresponding rules were thus not evaluated in this analysis.

In agreement with other reports [13], [22] we noticed that the predictive value of the weighted rules in Rega 8 was higher compared to the unweighted rules in Rega 5, however not significant. This may in part be due to the fact that NNRTIs and PIs, for which weighting factors were introduced, are less represented in our clinical data compared to the N(t)RTIs (Figure 1). This result corresponds with that of another study [20] in which the introduction of weighting did not change the GSS to a great extent.

Rega 8 had the highest sensitivity with respect to the other studied algorithms, although its specificity was the lowest (Table 3). This shift from specificity to sensitivity was a deliberate choice to extend the application of the Rega algorithm. Initially its purpose was solely to score resistance, so the previous versions of the Rega algorithm were heavily taking into account the genetic barrier to resistance associated with a large number of minor resistance mutations. In the latest version of Rega this has less effect. As a result, the focus of the algorithm shifted from predicting resistance and consequential therapy failure to predicting virological success in order to find an active, potent regimen. This change makes the Rega algorithm more in alignment with the other algorithms. How sensitivity and specificity should be balanced depends on the number of antiviral drugs that are still active. Therefore the Rega algorithm recommends different GSS cut-offs depending on the patient characteristics: 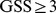 in case of therapy-naïve persons with no indications of transmitted drug resistance and in case of therapy-experienced persons, 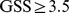 in case of therapy-naïve persons with indications of transmitted drug resistance and 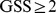 in case of therapy-experienced persons with limited treatment options. A higher risk for virologic failure and resistance development at mid-term can be expected when therapy changes with GSS below the target GSS of 3 are installed. Using this cut-off, Rega 8 reached a sensitivity score of 77.2% and a specificity score of 45.8% at 48 weeks follow-up, despite the fact that confounding factors like adherence, resistance- and treatment history, minor variants, baseline viral load and CD4 cell count were not taken into account. Kaplan-Meier analysis that was performed on the time to virological failure stratified by the GSS reported by Rega 8 showed that the Kaplan-Meier curves differed significantly between the group with a GSS of 2 and the group with a GSS of 3 (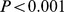) (results not shown). This supports the clinical relevancy of the Rega algorithm, despite the relatively small differences that were observed in comparison with the previously evaluated version. Regimens with 3 active drugs are required for long-term virologic response and are therefore currently recommended. However, virologic success with less potent regimens can be observed at the short- and mid-term time-points that were investigated in this analysis. As was in the pre-HAART era, when bi-therapies were prescribed [23], [24], some patients in our study still succeeded therapy at 48 weeks (22.8%, 116/507) although their GSS was 2.5 at the most. In contrast, regimens with 3 drugs lead to 48.5% (3430/7069) virological successes at 48 weeks. Three active drugs or a target GSS of at least 3 doubles the rate of success in the dataset. However, because genotypic interpretation systems have been used in clinical practice since 10 years, there is a bias towards a target GSS of 3 in the dataset (Figure 2). Therefore, not enough data is available in our to suggest that a target GSS of 2.5 would give similar success rates.

A previous report [14] that had as primary endpoint to evaluate the performance of the Rega algorithm (version 5.5) described the predictive value at 3 months. Viral load changes at that time point reflect more the activity of the treatment on the predominant virus population detected by baseline genotyping. However, clinicians are more interested in the prediction of longer-term virologic response (in our study up to 48 weeks), but then the outcome can be more influenced by the genetic barrier to resistance. We corrected (partly) for this bias by including information on treatment history as a surrogate for expected HIV drug resistance when calculating the performance of the algorithm.

In conclusion, we convincingly demonstrated that Rega 8 is significantly predictive for virological response and that additional variables should be taken into account to ensure an effective regimen. There was no distinguisable trend in the performance of the consecutive versions of the Rega algorithm, nor were there significant differences in AUC between Rega, ANRS and HIVdb. The clinical dataset used in this study may not have the power to show the gain of the sometimes minor improvements to excisting rules, of the specific rules for divergent subtypes, or of the rules for new drugs. Nevertheless, the reported analyses need to be done and published to give confidence to the users of the algorithms.
